# Unveiling the sound of the cognitive status: Machine Learning-based speech analysis in the Alzheimer’s disease spectrum

**DOI:** 10.1186/s13195-024-01394-y

**Published:** 2024-02-02

**Authors:** Fernando García-Gutiérrez, Montserrat Alegret, Marta Marquié, Nathalia Muñoz, Gemma Ortega, Amanda Cano, Itziar De Rojas, Pablo García-González, Clàudia Olivé, Raquel Puerta, Ainhoa García-Sanchez, María Capdevila-Bayo, Laura Montrreal, Vanesa Pytel, Maitee Rosende-Roca, Carla Zaldua, Peru Gabirondo, Lluís Tárraga, Agustín Ruiz, Mercè Boada, Sergi Valero

**Affiliations:** 1 Ace Alzheimer Center Barcelona, Universitat Internacional de Catalunya, Barcelona, Spain; 2 Networking Research Center on Neurodegenerative Diseases (CIBERNED), Instituto de Salud Carlos III, Madrid, Spain; 3 Accexible Impacto s.l., Urduliz, Bizkaia, Spain

**Keywords:** Alzheimer’s disease, Mild cognitive impairment, Early diagnosis, Neuropsychological tests, Machine Learning, Speech acoustics, Automated pattern recognition

## Abstract

**Background:**

Advancement in screening tools accessible to the general population for the early detection of Alzheimer’s disease (AD) and prediction of its progression is essential for achieving timely therapeutic interventions and conducting decentralized clinical trials. This study delves into the application of Machine Learning (ML) techniques by leveraging paralinguistic features extracted directly from a brief spontaneous speech (SS) protocol. We aimed to explore the capability of ML techniques to discriminate between different degrees of cognitive impairment based on SS. Furthermore, for the first time, this study investigates the relationship between paralinguistic features from SS and cognitive function within the AD spectrum.

**Methods:**

Physical-acoustic features were extracted from voice recordings of patients evaluated in a memory unit who underwent a SS protocol. We implemented several ML models evaluated via cross-validation to identify individuals without cognitive impairment (subjective cognitive decline, SCD), with mild cognitive impairment (MCI), and with dementia due to AD (ADD). In addition, we established models capable of predicting cognitive domain performance based on a comprehensive neuropsychological battery from Fundació Ace (NBACE) using SS-derived information.

**Results:**

The results of this study showed that, based on a paralinguistic analysis of sound, it is possible to identify individuals with ADD (F1 = 0.92) and MCI (F1 = 0.84). Furthermore, our models, based on physical acoustic information, exhibited correlations greater than 0.5 for predicting the cognitive domains of attention, memory, executive functions, language, and visuospatial ability.

**Conclusions:**

In this study, we show the potential of a brief and cost-effective SS protocol in distinguishing between different degrees of cognitive impairment and forecasting performance in cognitive domains commonly affected within the AD spectrum. Our results demonstrate a high correspondence with protocols traditionally used to assess cognitive function. Overall, it opens up novel prospects for developing screening tools and remote disease monitoring.

**Supplementary information:**

The online version contains supplementary material available at 10.1186/s13195-024-01394-y.

## Introduction

The digitization in the healthcare field experienced in the last few years has led to the emergence of new technologies with great potential for Alzheimer’s disease (AD) management [1]. This neurodegenerative disease is the most frequent type of dementia worldwide. With no effective treatment yet available, AD poses a significant challenge to the sustainability of existing healthcare systems [2]. As a result, the development of tools capable of detecting the disease in its early stages has become one of the most active research areas [3].

From a neuropsychological standpoint, AD typically manifests with impairments in memory, attention, language, executive and visuospatial functions, and behavior [2]. These alterations worsen as the disease progresses, eventually limiting the individual’s independence. At the pathophysiological level, AD is characterized by a cascade of brain-level events, including the accumulation of amyloid-$\beta$ plaques (A$\beta$), the formation of hyperphosphorylated tangles of tau protein (p-tau), and neuroinflammation [2, 4]. These neuropathological signatures ultimately lead to atrophy, decreased brain metabolism, and disruptions in brain connectivity, which cause the observed cognitive alterations [3–5].

In this context, it is well known that the entire landscape of neurological events in AD initiates several years before the onset of clinical symptoms and progresses silently until the first cognitive changes emerge [6]. Consequently, numerous diagnostic criteria have been proposed, incorporating information from biomarkers such as A$\beta$ and p-tau, measured by positron emission tomography (PET) or detected on cerebrospinal fluid (CSF), along with evidence of neurodegeneration assessed using magnetic resonance imaging (MRI) [4]. However, the detection of biomarkers through neuroimaging or lumbar puncture requires specialized equipment and trained personnel, rendering these procedures expensive and inaccessible for most healthcare centers. Furthermore, most patients consult a memory unit when their cognitive impairment is already evident and only a minority when the initial neuropsychological symptoms arise [2]. Collectively, these factors indicate that while these techniques have significant value for disease diagnosis, their use as population screening tools to identify individuals at risk of developing AD is limited.

Conversely, neuropsychological batteries have traditionally served as the initial assessment protocol for suspected cognitive impairment associated with AD [7, 8]. Nevertheless, these batteries still rely on the presence of clinicians in a specialized memory unit, making it a time-consuming procedure. Recognizing these limitations, numerous online abbreviated protocols have been proposed [1, 9]. Among these, neuropsychological assessment through the spontaneous speech (SS) analysis represents one of the most promising approaches [10]. Previous research supports the presence of language deficits in AD patients several years before the progression to the dementia stage, rendering it a valuable tool for detecting individuals in the early stages of mild cognitive impairment (MCI) [11, 12]. Additionally, language abilities exhibit associations with other cognitive domains such as memory, attention, and executive functions, suggesting that SS analysis has the potential to offer an approximate representation of an individual’s cognitive performance [12].

Moreover, the increasing interest in speech analysis using voice recordings stems from the versatility and abundance of information that can be extracted from this type of data. By employing modern natural language processing (NLP) techniques applied to automatic transcriptions [13] or based on the information derived from the raw waveform [14], it is possible to obtain information of great interest for assessing the cognitive performance of an individual. Within this wealth of information, the physical-acoustic features drawn directly from the raw sound represent an agnostic, standardized, and widely available resource [15, 16]. These features encompass parameters such as formants, pitch, and prosody, which are affected in numerous neurodegenerative diseases and affective disorders [15].

To date, most studies analyzing SS using Machine Learning (ML) techniques have focused on developing diagnostic tools to differentiate clinical phenotypes within the AD continuum. For instance, different research groups have shown that it is possible to obtain accuracies ranging from 80 to 90% for discriminating between healthy controls (HC) and AD dementia (ADD) using speech-derived information [17–22]. Other authors have extended their efforts beyond distinguishing ADD and HC, aiming to identify individuals in the early stages of MCI. In this context, results vary considerably based on the technique or sample used, with accuracies from 65 to 80% [18, 21, 23–25]. In contrast, very few authors have focused on addressing the relationship between SS and other aspects of the disease [26, 27]. For example, the investigation of the connection between SS and neuropsychological impairment has received limited attention [10, 28]. This relevant aspect is not widely available since demonstrating that SS can be a reliable proxy for an individual’s cognition requires its evaluation against standardized neuropsychological measures. However, most research has only utilized the Mini-Mental State Examination (MMSE) to explore the association of SS with cognitive status [29–31], typically within the context of the ADReSS challenge [32]. Moreover, the sample sizes employed, usually consisting of a few hundred subjects, severely restrict the ability to draw conclusive findings regarding the expected performance of the models [28].

The present study aims to extend previous research investigating how information obtained from a paralinguistic analysis of various SS tests can differentiate among clinical phenotypes and predict cognitive performance. For this purpose, firstly, we applied ML techniques to differentiate individuals with preserved cognition (subjective cognitive decline, SCD), patients with MCI, and those with ADD. Secondly, we developed models to predict changes in cognitive performance based on SS over the neuropsychological domains of memory, attention, visuospatial and executive functions, and language. As input variables for the models, we focused on information extracted from SS using standardized physical acoustic features obtained from the extended Geneva Minimalistic Acoustic Parameter Set (eGeMAPS) [15]. Compared to previous works, our study utilizes a significantly larger population from a real-world setting, comprising SCD individuals, and patients with MCI, and ADD. In addition, as a novelty, our research delves into the connection between speech and changes in cognitive domain performance across the AD spectrum using ML and physical-acoustic features.

## Methods

### Study participants

This study comprised 1500 individuals who underwent evaluation at the Memory Clinic of Ace Alzheimer Center Barcelona (Ace) (single site) between March 2022 and April 2023. The participants were referred to the Memory Clinic either by their General Health practitioner due to subjective cognitive complaints, or they attended the Open House Initiative without a previous referral from a physician [33]. All subjects completed neurological, neuropsychological, and social evaluations at Ace. The cognitive assessment included the Spanish version of the Mini-Mental State Examination (MMSE) [34], the memory test of the Spanish version of the 7 Minute screening neurocognitive battery [35], the short form of the Neuropsychiatric Inventory Questionnaire (NPI-Q) [36], the Hachinski’s Ischemia Scale [37], the Blessed Dementia Rating Scale [38], the Clinical Dementia Rating (CDR) [39], and the complete Neuropsychological Battery of Fundació Ace (NBACE) [7]. The final diagnosis for each participant was determined through consensus by a multidisciplinary team, including neurologists, neuropsychologists, and social workers, at a consensus diagnostic conference [40].

The 1500 participants included 135 individuals with SCD (CDR = 0) with no objective functional or cognitive impairments [41], 826 patients with MCI (CDR = 0.5) [42], and 539 with ADD (CDR > 0.5) [43]. Within the subjects with ADD, 398 had mild dementia (CDR = 1), and 141 had moderate dementia (CDR = 2). Table 1 summarizes the clinical and sociodemographic characteristics of the sample used for this study.

**Table 1:** Clinical and sociodemographic variables of the sample used for this study

Variable	All sample	SCD	MCI	ADD
Sample size	1500	135	826	539
Age (mean (SD))	76.17 (9.41)	67.06 (10.42)	74.40 (9.01)	81.15 (6.67)
Sex (% female)	63.47	63.70	62.23	65.31
Years of formal education (mean (SD))	8.58 (4.56)	12.30 (3.79)	8.61 (4.42)	7.62 (4.48)
MMSE (mean (SD))	25.18 (3.94)	29.33 (0.88)	26.85 (2.60)	21.62 (3.36)

Abbreviations: *SCD*, subjective cognitive decline; *MCI*, mild cognitive impairment; *ADD*, Alzheimer’s disease dementia; *MMSE*, Mini-Mental State Examination; *SD*, standard deviation

### Ethical considerations

This study and its informed consent were approved by the ethics committees of the Hospital Universitari de Bellvitge (Barcelona) (ref. PR007/22) under Spanish biomedical laws (Law 14/2007, 3 July, regarding biomedical research; Royal Decree 1716/2011, 18 November) and followed the recommendations of the Declaration of Helsinki. All participants signed an informed consent for the SS protocol.

### Acquisition and preprocessing of speech data

The SS protocol was carried out using the acceXible platform on a tablet. The assessments were conducted in Spanish within a calm and controlled environment. Initially, the participants were presented The Cookie Theft Picture and were instructed to provide a comprehensive description of the image [44]. Subsequently, they were given one minute to name as many different animals as possible. The participants’ voices were recorded during the administration of these two tests, and the collected data was utilized for further analyses. The average duration of the protocol was 109.07 ± 15.54 s, with a minimum and maximum duration of 74.6 and 158.9 s respectively.

The audio recordings were standardized to a frequency of 16 KHz, removing initial and final silent portions and applying the noise reduction model presented in [45] to eliminate potential environmental artifacts. The resulting audio data were manually reviewed. Then, features were extracted from the standardized feature set eGeMAPS [15] using the Python interface of the open-source toolkit OpenSMile [46]. The set of features from the eGeMAPS are oriented to provide a simplified and standardized selection of relevant acoustic parameters for detecting physiological changes in voice production guided by findings of previous related studies [15]. Based on prior research [19, 20], we adopted the default configuration provided by the OpenSMILE library [15, 46]. The variables were calculated from the eighteen low level descriptors using a symmetric moving average filter of three frames long. For a more comprehensive understanding of the computation of the paralinguistic variables, readers are directed to [15]. In total 176 variables were extracted from the voice recordings.

### Calculation of cognitive composites

Neuropsychological composites created from the NBACE battery were used to determine the cognitive status of participants. Composite scores are a widely employed approach to capture common factors of variance across different neuropsychological tests. Their purpose is to simplify the information by eliminating redundancy between variables offering a more comprehensive characterization of the cognitive domain being assessed [47]. In this study, five cognitive domains, typically examined in AD [48, 49], were considered: memory, attention, visuospatial functions, executive functions, and language. Structural equation models (SEM) were used to calculate the composite scores based on the neuropsychological structure described in [7], and defined by an expert panel of neuropsychologists.

Briefly, the SEM framework defines a measurement model in which observed items $\varvec{y} \in \mathbb {R}^i$ (e.g., *i* neuropsychological tests) are determined by unobservable factors $\varvec{\eta } \in \mathbb {R}^p$ (e.g., *p* higher cognitive functions) according to $\varvec{y} = \varvec{\upsilon } + \Delta \cdot \varvec{\eta } + \varvec{\epsilon }$, where $\varvec{\upsilon } \in \mathbb {R}^i$ corresponds to the intercepts of the regression paths, $\Delta \in \mathbb {R}^{i \times p}$ to the measurement slopes, and $\varvec{\epsilon } \in \mathbb {R}^i$ to the residual error. Additionally, the measurement model is subjected to a structural part that defines the relationship between observable and latent variables $\varvec{\eta } = \alpha + B \cdot \varvec{\eta } + \varvec{\zeta }$, where $\varvec{\alpha } \in \mathbb {R}^p$ is a parameter vector, $B \in \mathbb {R}^{p\times p}$ is a non-singular matrix with $\text {diag}(B) = 0$ indicating the relationship between latent variables, and $\varvec{\zeta } \in \mathbb {R}^p$ represents the latent variable residuals.

In the present study, the model parameters were adjusted using the robust maximum likelihood estimator, and the variances of the latent variables were fixed to 1 for model identification (unit variance method [47]). The model coefficients were estimated considering the baseline evaluations of the entire Ace database (*N *= 23,987)―including individuals HC/SCD, MCI, and ADD―using the R package lavaan [50]. The composite scores were adjusted for age, sex, and years of formal education effects using linear regression models. Further details on the composites and their calculation can be found in the Appendix A.

The memory composite was created considering the variables long-term and recognition memory of the Word List subtest from the Wechsler Memory Scale, third version (WMS-III) [51]. The attention composite included the Digit Forward and Digit Backwards from the Wechsler Adult Intelligence Scale, Third Edition (WAIS-III) [52]. To define the visuospatial functions, the 15-Objects Test [53], the Poppelreuter-type overlap figures [54], and the Luria’s Clock test [55] were considered. The executive functions were calculated from the Phonetic and Semantic Verbal fluencies [56, 57] and the Automatic Inhibition subtest of the Syndrom Kurtz Test (SKT) [58]. Finally, language function included the abbreviated 15-item naming test from the Boston Naming Test (BNT) [59] and the Verbal Comprehension and Repetitions [7].

### Machine Learning modeling

In the present study, two different problems were addressed. Firstly, classification models were developed to differentiate between clinical phenotypes. Secondly, regression models were implemented to predict the cognitive composites outlined in the “Calculation of cognitive composites” section. The models used in each problem are described below.

For the classification problems, the following models were used: random forest (RF), extreme gradient boosting (XGB), support vector machine (SVM), and k-nearest neighbor (KNN). Due to the high dimensionality of the input data, the SVM and KNN algorithms were combined with a previous feature selection step. The feature selection aims to identify the optimal combination of variables by eliminating those that are irrelevant/redundant. We performed feature selection using a wrapper-based approach involving two sequential stages: candidate subset generation (SG) and subset evaluation (SE) [60]. For the SG component, we utilized genetic algorithms (GA), a population-based metaheuristic optimization strategy inspired by the natural selection process [61]. For the SE, we considered the mean balanced accuracy ($\text {BA} = 0.5 \cdot [\text {sensitivity} + \text {specificity}]$) obtained through fivefold cross-validation (CV) from predictions derived from a Gaussian naive Bayes classifier. Feature selection was not applied for the RF and XGB models because these types of algorithms, not based on distance like SVM or KNN, are more robust to the high dimensionality.

Moreover, the hyperparameters for RF, XGB, and SVM models were determined using a hyperparameter optimization (HPO) framework. A nested ten-fold CV was applied to the training set to perform the HPO. The HPO was implemented using the Optuna open-source library [62], applying a Bayesian optimization (BO) using a tree-structured Parzen estimator (TPE) as a surrogate model [63]. Since the KNN model had a small number of hyperparameters, they were optimized using a grid search. In addition, given the high-class imbalance in the classification problems, the KNN was combined with the synthetic minority over-sampling technique (SMOTE) applied to the minority class [64].

For the regression tasks, the same models adapted for predicting quantitative variables (RF, XGB, GA-SVM, and GA-KNN) were used. In this case, the SE step of the feature selection strategy used in the GA-SVM and GA-KNN was performed using a KNN regressor minimizing the mean squared error (MSE). For the BO-TPE, the MSE evaluated by a nested tenfold CV applied on the training set was minimized. The optimized hyperparameters for each model and the configuration details for BO-TPE and the GAs are provided in Appendix B.

All models were implemented using Python (v3.9.16). The scikit-learn library was utilized for RF, KNN, and SVM algorithms [65]. The XGB package [66] was employed for the XGB models. Finally, for the GAs, the home-made library pywinEA2 available on GitHub was used.

### Experimental setup

Among the main objectives of this study was to differentiate clinical phenotypes. For this purpose, the following problems were addressed: differentiation of individuals with a preserved cognitive state (SCD) from those with cognitive impairment (MCI and ADD), discrimination between SCD and ADD, classification of MCI and ADD, and the distinction between SCD and MCI. For the prediction of the cognitive composites, models were fitted on each of the five composites mentioned in the “Calculation of cognitive composites” section.

**Fig. 1:**
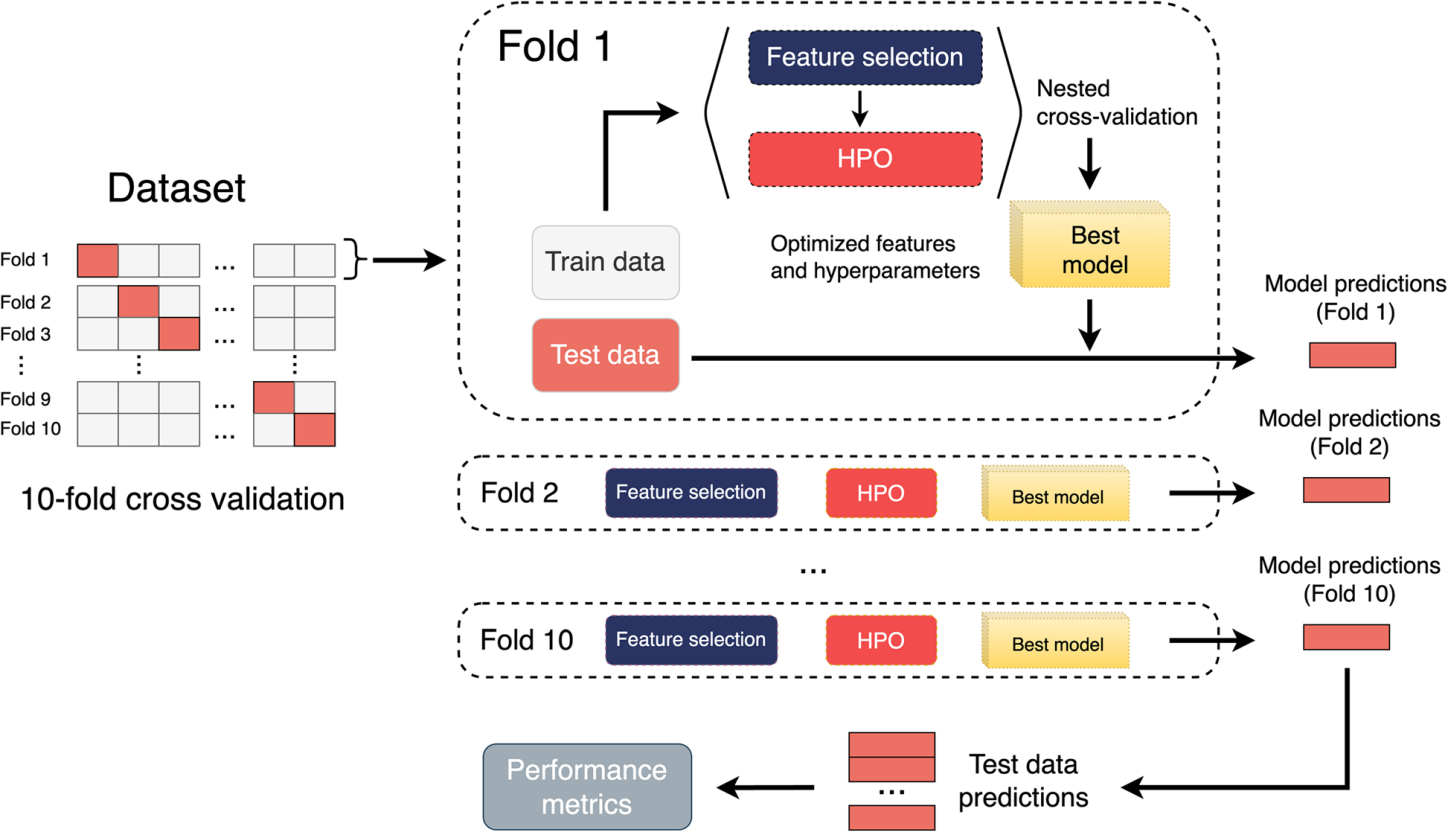
Pipeline applied to train and evaluate the different models used in this study. The input data were divided into ten folds according to a cross-validation (CV) scheme. Feature selection and hyperparameter optimization (HPO) were conducted using a nested CV applied to the training data. The resulting model from this nested CV was then used to make the predictions on the test set. The final performance metrics were calculated based on the average performance of the predictions obtained on the test set

All models were evaluated using a ten-fold CV. Performance metrics were reported as the mean values obtained on the test set. The HPO and feature selection techniques, as described in the “Machine Learning modeling” section, were implemented using a nested CV approach on the training set to prevent overfitting. For classification tasks, CV was conducted with class stratification. Figure 1 illustrated the training and model validation pipeline applied for all the algorithms.

The following performance metrics were considered for the classification problems:

1 $$\begin{aligned} \text {Sensitivity} = \frac{\text {TP}}{\text {TP} + \text {FN}}\;, \end{aligned}$$

2 $$\begin{aligned} \text {Specificity} = \frac{\text {TN}}{\text {TN} + \text {FP}}\;, \end{aligned}$$

3 $$\begin{aligned} \text {Precision} = \frac{\text {TP}}{\text {TP} + \text {FP}}\;, \end{aligned}$$

4 $$\begin{aligned} \text {Balanced accuracy (BA)} = \frac{\text {Sensitivity} + \text {Specificity}}{2}\;, \end{aligned}$$

5 $$\begin{aligned} \text {F1-score (F1)} = 2 \cdot \frac{\text {Precision} \cdot \text {Sensitivity}}{\text {Precision} + \text {Sensitivity}}\;, \end{aligned}$$

where TP and TN represent true positives and negatives and FN and FP stand for false positives and negatives. For the regression problems, the correlation coefficient between model predictions ($\hat{Y} \in \mathbb {R}^n$) and true values ($Y \in \mathbb {R}^n$) was considered, as well as the following metrics:

6 $$\begin{aligned} \text {Mean absolute error (MAE)} = \frac{1}{n} \cdot \sum _{i = 1}^n |Y_i - \hat{Y}_i| \;, \end{aligned}$$

7 $$\begin{aligned} \text {Explained variance (EV)} = 1 - \frac{\text {Var}[\,Y - \hat{Y}\,]}{\text {Var}[\,Y\,]} \;, \end{aligned}$$

8 $$\begin{aligned} \text {Relative MEA (RMEA)} = \frac{\text {MAE}}{|P_{95\%}^{v} - P_{5\%}^{v}|} \;, \end{aligned}$$

with $\text {Var}[\,\cdot \,]$ being the variance and $|P_{95\%}^{v} - P_{5\%}^{v}|$ representing the range of the variable *v* between the 95% and 5% percentiles. Consequently, the RMEA allows contextualizing the magnitude of the MEA in relation to the magnitude of the analyzed variable.

As input variables for all models, the 176 variables extracted from the voice recordings (the “Acquisition and preprocessing of speech data” section) and sociodemographic variables including age, years of education, and sex were considered. For the distance-based models such as SVM and KNN, the variables were standardized to z-scores based on the statistics of the training data.

All experiments were conducted on the Ace computing’s cluster, composed of 368 CPU cores and 1280 GB of RAM running on Rocky Linux OS (v8.6).

## Results

### Cognitive composites analysis

As mentioned in the “Calculation of cognitive composites” section, five cognitive composites were derived from the neuropsychological variables of the NBACE [7]. These composites encompassed the cognitive domains of attention, executive functions, language, memory, and visuospatial functions. The SEM used for their estimation showed good fit indices supporting the consistency of the proposed factorial structure (comparative fit index = 0.93, Tucker-Lewis index = 0.92, mean square error of approximation = 0.07) [47].

**Fig. 2:**
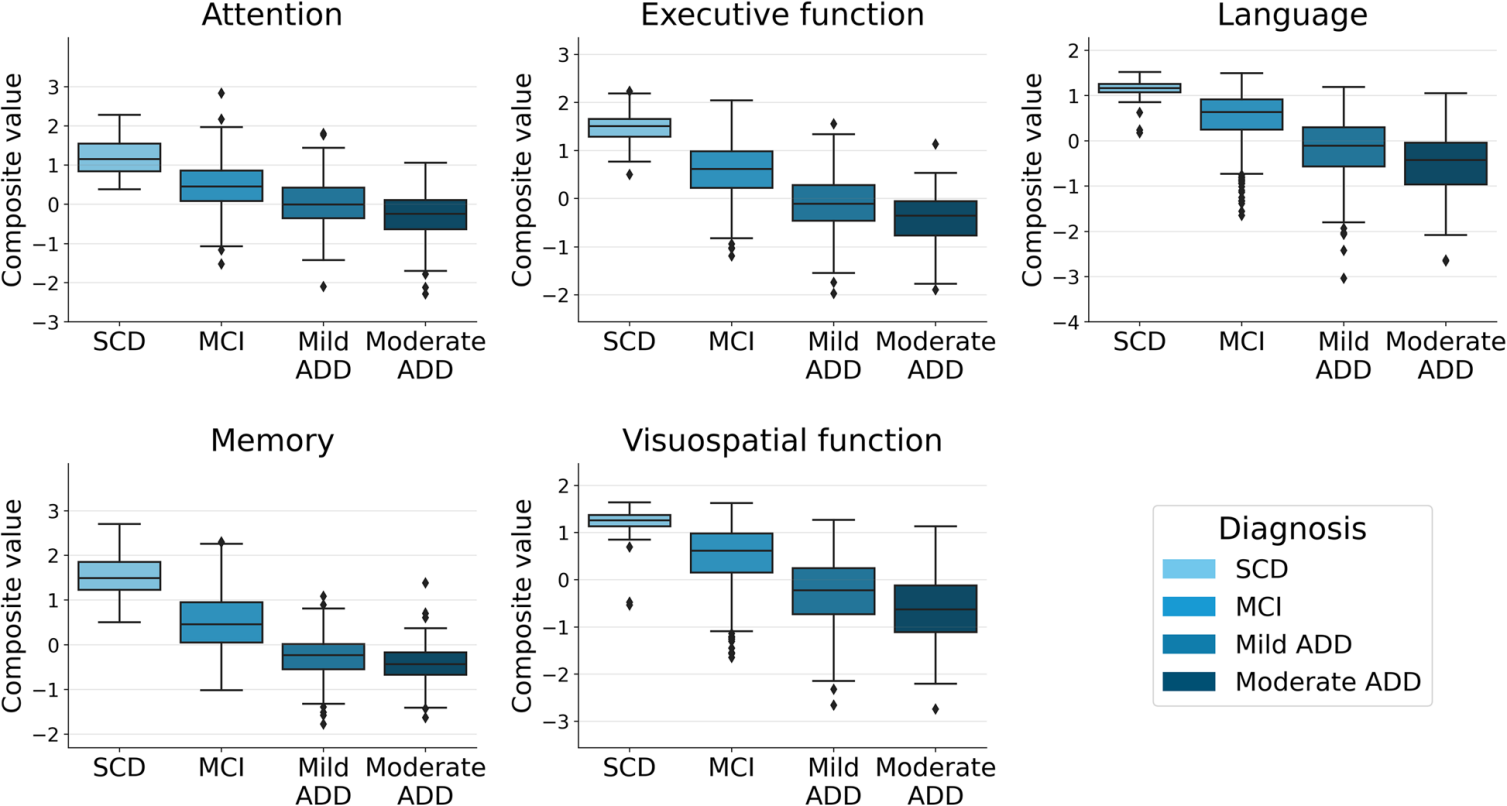
Cognitive composite values were obtained through structural equation models (SEM) and adjusted for age, sex, and years of education. The scores were represented based on the diagnostic group. Abbreviations: SCD, subjective cognitive decline; MCI, mild cognitive impairment; ADD, Alzheimer’s disease dementia

Figure 2 illustrates the composite values based on clinical diagnosis. Using linear regression models, significant differences in all composite scores across different diagnostic groups were found (Bonferroni adjusted *p*-value < 0.05) (refer to Appendix C for further details). Across all cognitive domains, as expected, SCD subjects exhibited the highest composite scores, while individuals with MCI displayed intermediate ones, and the ADD group showed the lowest composite values.

Table 2 presents the discriminatory performance achieved using a logistic regression model for each composite. The mean values obtained on the test set from ten repetitions of ten-fold CV are displayed. As expected, all composites exhibited a strong discriminatory ability between SCD and ADD individuals. They also offered a clear, although lower, differentiation of SCD and MCI subjects. Notably, the composites of executive functions, language, memory, and visuospatial functions provided the best discrimination between SCD and ADD individuals (AUC > 0.98). The attention composite showed a lower predictive performance (AUC $\approx$ 0.95). In the detection of MCI individuals, the values were slightly lower. The executive function composite showed the highest predictive capacity (AUC $\approx$ 0.93). The visuospatial, language, and memory composite scores also demonstrated good discriminatory ability (AUC > 0.90). The attention composite showed the poorest performance (AUC $\approx$ 0.84).

**Table 2:** Discriminatory capacity of cognitive composites for differentiating clinical phenotypes using subjects with subjective cognitive decline (SCD) as reference

Composite	Accuracy		Sensitivity		Specificity		AUC	
	ADD	MCI	ADD	MCI	ADD	MCI	ADD	MCI
Attention	0.87	0.74	0.86	0.74	0.89	0.75	0.95	0.84
Executive function	0.97	0.84	0.97	0.83	0.98	0.91	0.99	0.93
Language	0.96	0.81	0.95	0.79	0.98	0.91	0.99	0.91
Memory	0.98	0.81	0.98	0.80	0.97	0.87	0.99	0.90
Visuospatial function	0.95	0.79	0.95	0.77	0.98	0.92	0.99	0.91

Mean value obtained over the test set from ten repetitions of tenfold cross-validation. A logistic regression model was employed to predict clinical phenotypes. The composite scores were considered as independent variables

Abbreviations: *AUC*, area under the curve; *MCI*, mild impairment; *ADD*, Alzheimer’s disease dementia

### Spontaneous speech for differentiating clinical phenotypes

**Fig. 3:**
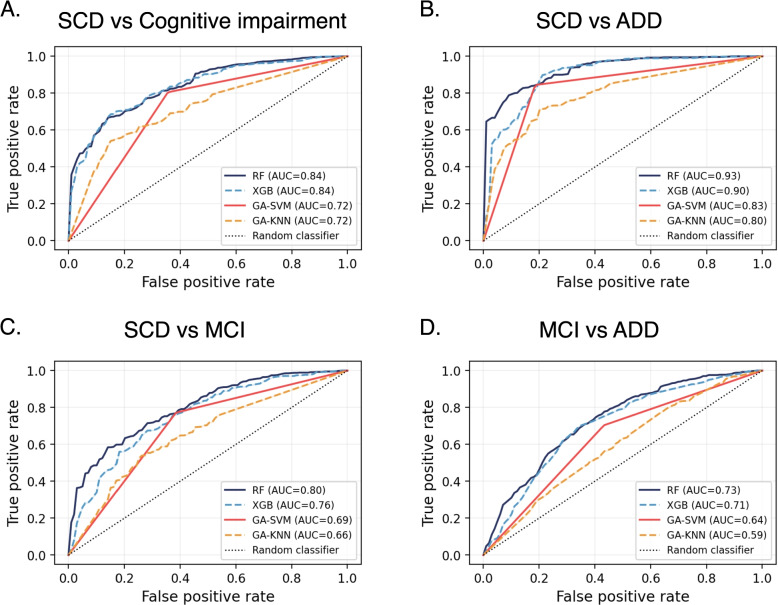
The average values of the receiver operating characteristic (ROC) curves, obtained for the models discussed in the “Machine learning modeling” section, are presented. The ROC curves were calculated on the test set from a ten-fold cross-validation. The analyzed classification tasks encompassed: **A** discrimination between subjective cognitive decline (SCD) and patients with mild cognitive impairment (MCI) or Alzheimer’s disease dementia ADD, **B** SCD vs ADD, **C** SCD vs MCI, **D** and MCI vs ADD. Abbreviations: RF, random forest; XGB, extreme gradient boosting; GA, genetic algorithm; SVM, support vector machine; and KNN, k-nearest neighbor

The results of the top-performing models for distinguishing clinical phenotypes are presented in Table 3. Due to significant imbalances between classes, the models with the highest F1-score value were selected. The receiver operating characteristic (ROC) curves for each problem, incorporating all algorithms, are depicted in Fig. 3. For the models that integrated GAs, the selected variables are detailed in the Supplementary material. Appendix E provides a summary of the features that were consistently chosen by the GAs during the CV.

Regarding the algorithms implemented, the tree-based models showed the best performance for most of the problems. Specifically, the RF obtained the highest F1 for the differentiation between SCD and MCI/ADD, the XGB achieved the best results in SCD-ADD and MCI-ADD problems, and the GA-SVM model outperformed the rest of the algorithms for distinguishing SCD and MCIs. The GA-KNN combination consistently demonstrated the poorest performance across all the problems (refer to Appendix D for further details).

When distinguishing between SCD and subjects with cognitive impairment (MCI/ADD), an F1-score of 0.85 was achieved. In this comparison, sensitivity and specificity were close to 0.75. The performance notably improved when differentiating between SCD and ADD, reaching an F1-score of 0.92, with a sensitivity of approximately 0.90 and specificity of around 0.80. In contrast, in all cases involving the identification of MCI subjects, the performance decreased. When discerning between MCI and SCD, a specificity of 0.77 was reached, but at the expense of a high rate of false positives (sensitivity of 0.62). Moreover, the specificity for distinguishing ADD from MCI decreased to 0.71 while maintaining a low sensitivity (< 0.65).

Finally, we conducted a subanalysis excluding sociodemographic information and fitting the top-performing models based purely on physical-acoustic variables. In this scenario, we observed a decline in performance compared to the previous models. Specifically, for distinguishing SCD from individuals with cognitive impairment, the F1-score dropped to 0.80 (from 0.85). In the discrimination between SCD and ADD, the F1-score decreased to 0.81 (from 0.92). For identifying SCD and MCI, the F1-score was similar (0.78 vs 0.77). Finally, in the classification of MCI and ADD, the F1-score moved to 0.55 (from 0.63).

**Table 3:** Average values of the classification metrics computed on the test set for the models achieving the highest F1-score in each of the problems

Problem	Best model	BA	F1	Precision	Sensitivity	Specificity
SCD vs cognitive impairment	RF	0.75	0.85	0.97	0.76	0.73
SCD vs ADD	XGB	0.84	0.92	0.94	0.90	0.79
SCD vs MCI	GA-SVM	0.69	0.84	0.93	0.77	0.62
MCI vs ADD	XGB	0.67	0.63	0.56	0.72	0.63

Abbreviations: *BA*, balanced accuracy; *F1*, f1-score; *RF*, random forest; *XGB*, extreme gradient boosting; *GA-SVM*, genetic algorithm-support vector machine; *SCD*, subjective cognitive decline; *MCI*, mild cognitive impairment; *ADD*, Alzheimer’s disease dementia

### Spontaneous speech for predicting cognitive domains

**Fig. 4:**
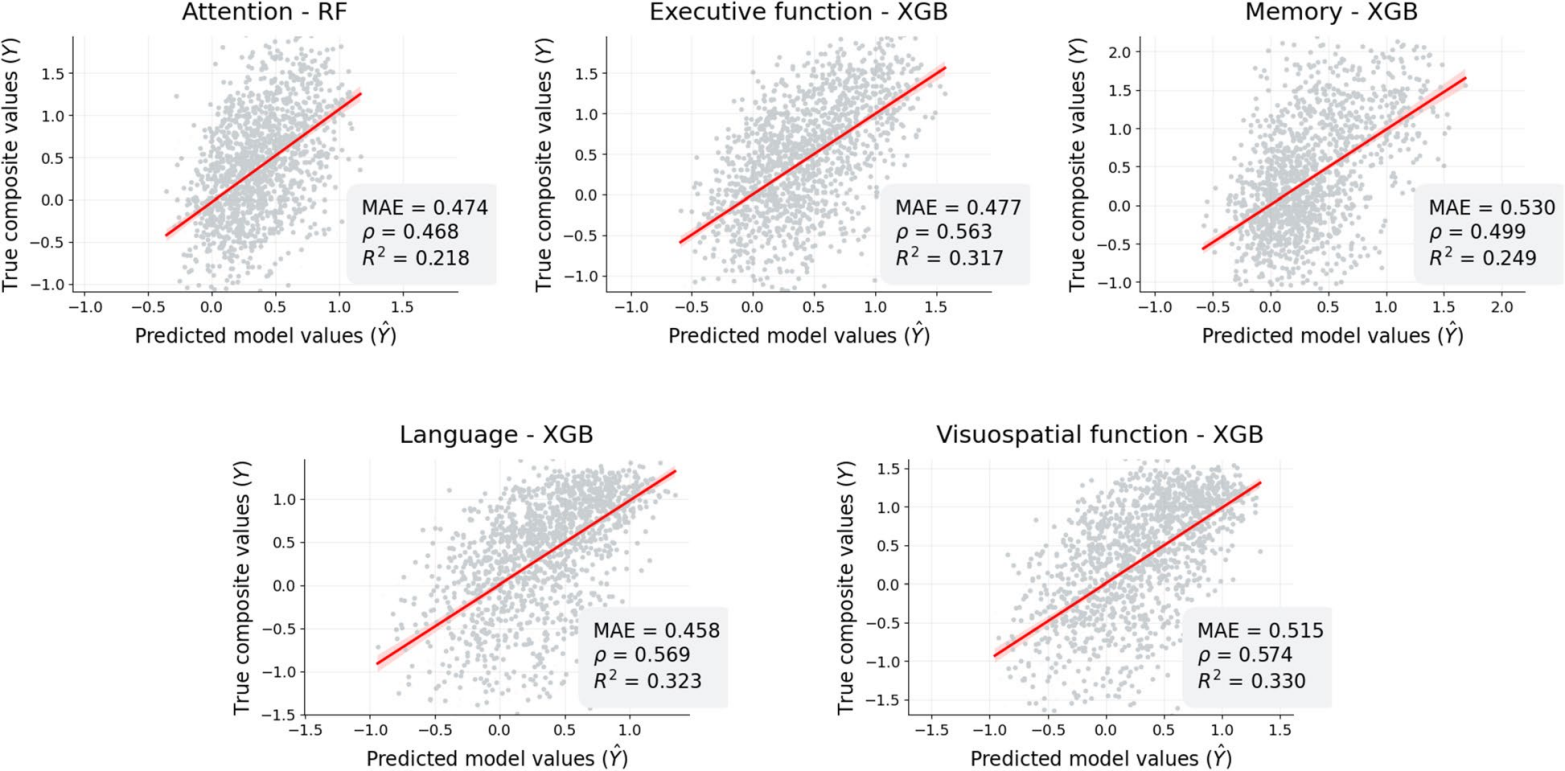
Correlation between the predicted values generated by the models and the actual values for each cognitive domain under examination. The predictions from the model with the lowest mean absolute error (MAE) were represented. The values depicted in the figure correspond to the predictions made on the test set from a ten-fold cross-validation. To minimize the influence of outliers in the representation, the scales of both the *X* and *Y* axes were adjusted considering the 95th percentile of the true values. Abbreviations: $\rho$, correlation between model predictions ($\hat{Y}$) and true values (*Y*); *R*^2^, coefficient of determination; RF, random forest; XGB, extreme gradient boosting

The predictions given by the best regression models used for estimating the five cognitive scores outlined in the “Calculation of cognitive composites” section, are collected in Fig. 4. Alternatively, the different regression metrics described in the “Experimental setup” section are presented in Table 4. The values of the former table were stratified by clinical phenotype.

Overall, the tree-based models exhibited superior performance. The RF model achieved the lowest MAE in predicting the attention score, while the XGB outperformed the other algorithms for predicting the remaining cognitive domains. Detailed results of all the models can be found in Appendix D. Furthermore, the variables selected by the GAs for the SVM and KNN models are listed in the Supplementary material and Appendix E.

**Fig. 5:**
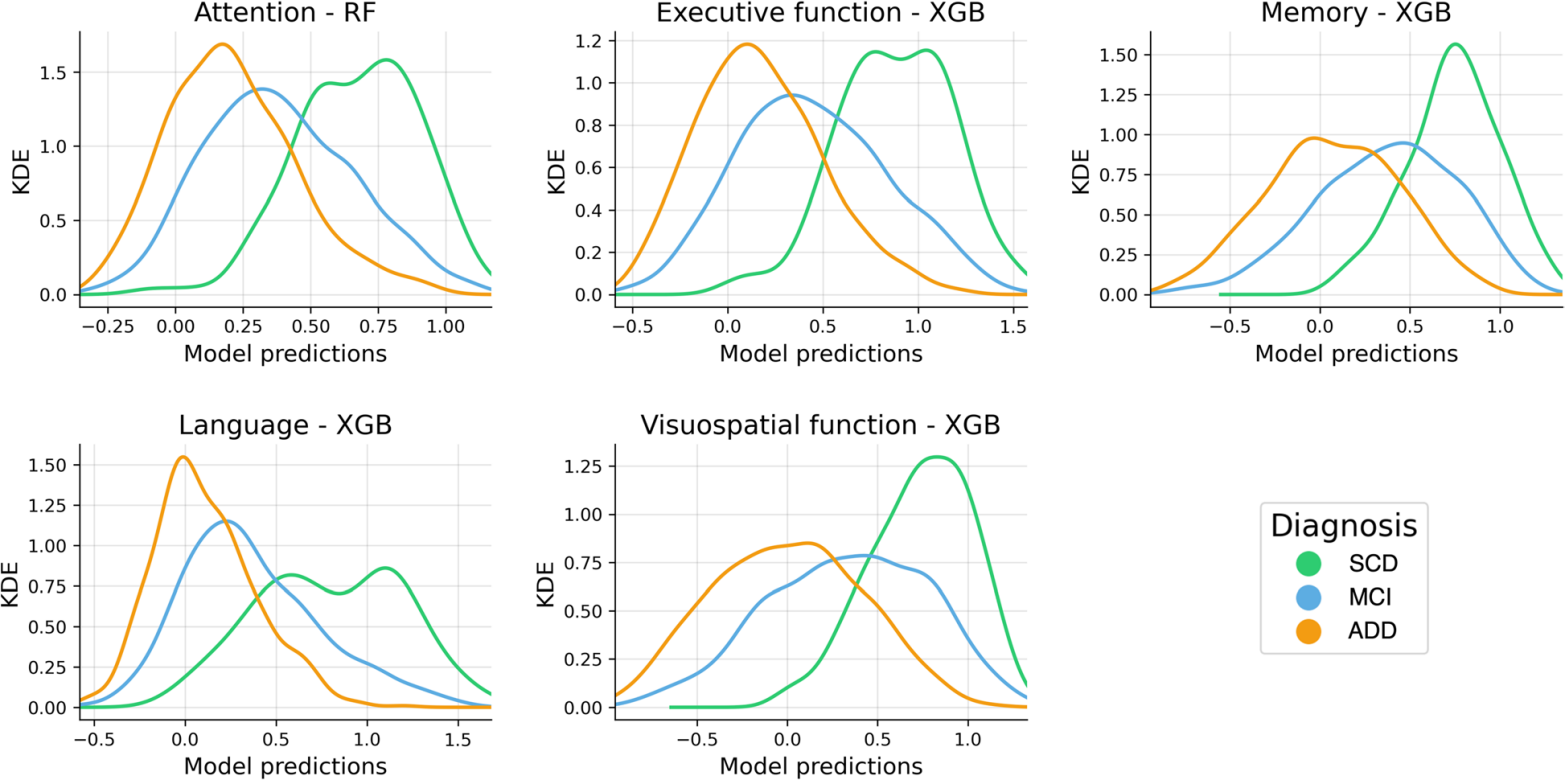
Distribution of predictions given by the models stratified by clinical diagnosis for each of the cognitive functions. The *Y*-axis represents a kernel density estimation (KDE) of the model predictions distribution (*X*-axis). Abbreviations: SCD, subjective cognitive decline; MCI, mild cognitive impairment; ADD, Alzheimer’s disease dementia; RF, random forest; XGB, extreme gradient boosting

A consistent correspondence between the model predictions and the actual values for each cognitive domain was observed. The language composite regression exhibited the best predictive performance, with a correlation coefficient of 0.57, an EV of 32.3%, and an RMAE of 17.8%. Similarly, for the executive and visuospatial functions, comparable results were achieved, with correlations above 0.56, EVs greater than 31.0% and RMAEs below 18.0%. The models also performed well for the memory and attention composites, although the correspondence between the predictions and the actual values decreased slightly (correlation < 0.5). When stratified by clinical diagnosis, the models performed better, with a fewer MAE, in subjects with MCI across all cognitive domains. The predictions for the SCD group showed the highest errors in attention, executive function, and memory composites. In contrast, the ADD group exhibited the highest error in the composites of language and visuospatial functions. Figure 5 illustrates the prediction distributions generated by the models stratified by the diagnostic group. The regression models predicted higher values in all the composites for the SCD, lower values for the MCIs, and a reduced estimate for the ADD individuals.

Similar to the classification problems (see the “Spontaneous speech for differentiating clinical phenotypes” section), we investigated the influence of demographic variables on the best models. For this purpose, we eliminated the variables of age, years of formal education, and sex from the models. In this context, we observed a marginal decline in their performance, while still maintaining correlations between predicted and true values that closely mirrored those obtained when demographic information was integrated. Specifically, for the attention composite, the correlation and EV were at 0.43 and 18.3%, respectively. Concerning executive function, the correlation and EV values shifted to 0.51 and 26.0%. The performance scores for the memory composite declined to 0.42 and 18.0%. For the language composite, the new values were 0.50 and 25.3%. Finally, for the visuospatial function, the correlation and EV dropped to 0.51 and 26.3% respectively.

**Table 4:** Regression metrics obtained by the best models in the prediction of each cognitive domain

Sample	Metric	Attention	Executive function	Language	Memory	Visuospatial function
All	RMAE (%)	18.007	17.421	17.801	18.330	17.880
	MAE	0.474	0.477	0.458	0.530	0.515
	Correlation	0.468	0.563	0.569	0.499	0.574
	EV^a^ (%)	21.778	31.696	32.345	24.924	32.964
SCD	RMAE (%)	22.063	23.295	16.736	26.588	18.401
	MAE	0.580	0.638	0.431	0.769	0.530
	Correlation	0.062	0.082	0.009	0.076	0.002
	EV^a^ (%)	–	–	–	–	–
MCI	RMAE (%)	16.282	15.339	15.573	17.023	16.125
	MAE	0.428	0.420	0.401	0.492	0.464
	Correlation	0.370	0.477	0.487	0.383	0.485
	EV^a^ (%)	12.494	17.759	17.559	10.321	18.114
ADD	RMAE (%)	19.634	19.141	21.483	18.265	20.440
	MAE	0.517	0.524	0.553	0.528	0.589
	Correlation	0.243	0.205	0.330	-0 036	0 347
	EV^a^ (%)	3.668	–	6.192	–	6.560

For each cognitive domain, the regression metrics obtained by the best models are presented. The random forest (RF) was the best model at predicting the attention score, while the extreme gradient boosting (XGB) performed better on all other scores. The metrics were calculated for the entire sample and stratified by clinical phenotype

Abbreviations: *MAE*, mean absolute error; *RMAE*, relative MAE (described in the “Experimental setup” section); *EV*, explained variance; *SCD*, subjective cognitive decline; *MCI*, mild cognitive impairment; *ADD*, Alzheimer’s disease dementia

^a^ The EV is not shown when the variance of the true values was lower than the variance of the residuals

## Discussion

The present study shows that the automated analysis of a brief SS test using ML techniques consistently detects cognitive alterations along the AD spectrum. To the best of our knowledge, this is the first study with a large sample from a clinical setting exploring the association between SS and cognitive performance across neuropsychological domains.

Firstly, several ML models were applied to distinguish clinical phenotypes associated with AD. Our models focused on differentiating between individuals diagnosed with SCD, MCI, and ADD. The results demonstrated a good differentiation between SCD individuals from those with already manifest cognitive impairment (MCI/ADD) (AUC = 0.84) as well as between SCD and ADD patients (AUC = 0.93) (Table 3). These findings are consistent with previous studies [17–22, 67–71]. For example, in [20], they achieved an accuracy of 80.28% on the test set for detecting subjects with dementia using information derived from *The Cookie Theft Picture* description task. In the same line, in [17], they reached an AUC of 0.86 for discriminating between HC and ADD subjects. Similarly, the authors of [21] obtained an AUC of 0.93 for distinguishing HC individuals from those with dementia and an AUC of 0.88 for differentiating dementia and non-dementia subjects. Therefore, our findings provide new evidence supporting the potential of SS for the identification of individuals with ADD.

In contrast, the models exhibited a moderate predictive performance for identifying individuals with MCI (MCI vs SCD: AUC = 0.80, MCI vs ADD: AUC = 0.73). These findings are consistent with the results previously reported in the literature [18, 21, 23–25]. In their study, [18] identified MCI subjects with a BA of 0.65, slightly lower than the results presented in this work (BA = 0.69, see Table 3). With subtly better performance, in [23], they used linguistic features extracted from transcripts, reporting a specificity and sensitivity of 78% and 74%, respectively. However, it is important to note that their study was supported by a considerably smaller sample size than ours, limiting conclusions about the model’s accuracy. Consistent with the findings of this work, in [21], they observed that the identification of subjects with MCI was notably more challenging compared to detecting dementia, obtaining an AUC of 0.74. Overall, the difficulties in identifying MCI stem from its inherent heterogeneity, encompassing different subtypes and potential underlying etiologies. Furthermore, the cognitive deficits in this population are subtle and can be influenced by factors such as age, education level, and individual cognitive abilities, often masking the underlying MCI status. Consequently, the boundary between normal cognition and MCI remains inherently fuzzy, limiting the performance of the models [42]. Future studies should strive to overcome these limitations and enhance the ability to identify the cognitive changes that emerge during the MCI stage. Nevertheless, our results, based exclusively on the physical-acoustic properties of the sound, demonstrate that the application of Artificial Intelligence (AI) techniques to SS data offers valuable insights for identifying patients with MCI.

This study also investigated the potential to estimate cognitive performance using the paralinguistic variables derived from SS tests. For this purpose, neuropsychological tests from a standardized battery of neuropsychological measures were grouped into five composite scores representing the cognitive domains of attention, executive functions, language, memory, and visuospatial functions [7]. These neurocognitive composites were the target variables of the ML models. As outlined in the “Cognitive composites analysis” section, the resulting composites discriminate between the different diagnostic groups. This discriminatory ability was expected, as the neuropsychological tests forming the composites are partially instrumental in defining the diagnoses. Nevertheless, these results show that their grouping into cognitive domains is consistent and effectively summarizes the information from the individual neuropsychological tests into higher cognitive functions. Therefore, this subanalysis supports the use of these neuropsychological groupings to describe the different cognitive functions analyzed.

The algorithms used to infer the neurocognitive composites based on the SS tests and physical-acoustic features showed a strong predictive ability (Fig. 4). In general, we observed that the models were proficient in predicting the different cognitive scores with a correlation close to 0.5 relative to the actual values (Table 4). Nevertheless, our findings indicated a significant increase in model errors within the SCD and ADD groups. For example, the correlation between model predictions and actual values in SCD subjects remained poor, likely due to the ceiling effect present in many of the neuropsychological tests used to construct the composites or an overrepresentation of MCI subjects within the sample. However, despite this, we observed a meaningful alignment of the model predictions with the different disease stages, associating higher values for SCD subjects, declining scores in the MCI stage, and lower estimates for ADD patients (Fig. 5). This result becomes especially relevant for the remote detection of cognitive impairment in the general population, as the SS test can be completed within an average duration of 110 s, and the correspondence between model predictions and disease stages is consistent. In addition, the increased predictive ability obtained for MCI patients (see Table 4) is particularly significant, as this stage holds great importance for developing screening tools, recruiting patients for clinical trials, and monitoring disease progression [2].

Regarding the models used, our results also provided insightful findings. First, we noted that tree-based algorithms, specifically RF and XGB, consistently surpassed distance-based algorithms (i.e., SVM and KNN) across tasks. We hypothesize that this superiority can be attributed to the high dimensionality of the input data and the robust nature of tree-based algorithms in handling non-smooth distributions [66, 72]. Moreover, despite incorporating a prior feature selection step to mitigate the sensitivity of distance-based algorithms to the high dimensionality, the GAs consistently selected a high number of variables. This elevated number of input features is presumably responsible for the poorer performance achieved by these models. It suggests that the effectiveness of feature selection techniques may not scale optimally with the increasing number of input variables [60].

On the other hand, we observed that excluding demographic variables from the models harmed their performance. These findings underscore the potential advantages of incorporating contextual information about the patient in SS and AI-based screening tools, enabling the algorithms to uncover nonlinear interactions among input variables [19, 21]. For instance, it is reasonable to expect that a specific response pattern on an SS test has distinct implications for an older non-literate individual compared to a young person with a high level of education. However, additional investigation is needed to evaluate the standalone predictive capacity of SS and the consequences of integrating contextual information into the models. In this regard, special attention should be directed towards variables that can be readily and efficiently collected through a remotely administered protocol, such as self-reported information on comorbidities or a family history of neurodegenerative diseases.

Our study shows that an automated analysis of speech based on paralinguistic features and ML techniques has the potential for detecting and assessing AD stages. Compared to other modalities, such as neuroimaging or plasma biomarkers, SS-based protocols represent a fast, cost-effective, and accessible tool for evaluating the patient’s cognitive status despite lower diagnostic accuracy [10, 73]. The SS protocol is non-invasive, does not require expensive equipment or highly trained personnel, and is a patient-friendly procedure. Furthermore, beyond the implementation of SS as an early screening tool, periodic assessment of SS could provide valuable insights into the decline in different cognitive domains on a simple and rapid basis. Overall, it opens up new opportunities for implementing novel and widely accessible to the general population screening strategies.

From a methodological standpoint, our study benefits from using standardized SS tests and paralinguistic features, facilitating the replication of our findings. Moreover, unlike previous studies, we developed models capable of inferring cognitive performance from SS data using a large sample of subjects at different disease stages. In addition, we employed optimized and transparently evaluated ML models, providing detailed fit indices that enable easy comparison with other studies. Collectively, our work presents a promising avenue for leveraging automated speech analysis in AD, offering potential benefits for the early detection and monitoring of cognitive decline.

Nevertheless, this study has certain limitations. Firstly, despite using a large sample of subjects, especially compared with most current studies [17, 19, 20, 23, 68, 71], the generalization of our results should be confirmed by future prospective analyses involving larger samples and individuals from different cohorts and in different languages. This recurrent issue is commonly encountered in studies employing ML techniques and represents a significant challenge for potential translation to the clinical setting. Secondy, our study was conducted using data generated in a controlled clinical environment, ensuring the acquisition of high-quality audio data. However, the remote administration of the SS protocol is expected to introduce noise and other factors that may potentially impact the performance of the models. Consequently, the evaluation of the effectiveness of the proposed approach with remotely generated data will be an aspect of interest to be explored in future studies. Moreover, our study relies solely on a restricted set of paralinguistic features. As demonstrated by other researchers [18, 19, 21], using more diverse data derived from speech analysis, such as employing NLP techniques [13], could substantially enhance the performance of predictive models. For future investigations, it may be worthwhile to consider including broader and more diverse SS parameters to improve the performance established in this study. Finally, this work has a cross-sectional design due to the restricted access to follow-up information. Future research endeavors will be necessary to explore how longitudinal analysis of SS data can provide relevant insights into predicting aspects related to the AD continuum, and differentiate ADD from other types of dementia.

## Conclusion

In conclusion, the convergence of Artificial Intelligence advancements and the rapid digitization witnessed in recent years has set the stage for developing new technologies capable of monitoring AD in a simple and increasingly accessible manner. Among these innovative technologies, SS protocols, such as the one employed in this study, stand out. These technical breakthroughs hold great potential in enabling early and precise detection of cognitive changes within the AD continuum, ultimately facilitating remote access to specialists and personalized therapies. Our study provides new evidence to the field, demonstrating the feasibility of inferring cognitively impaired performance across different cognitive functions from SS data, establishing a solid foundation for future developments in predictive models.

### Supplementary Information

**Additional file 1.** Supplementary material associated with this article is provided.

## Figures and Tables

**Figure:**
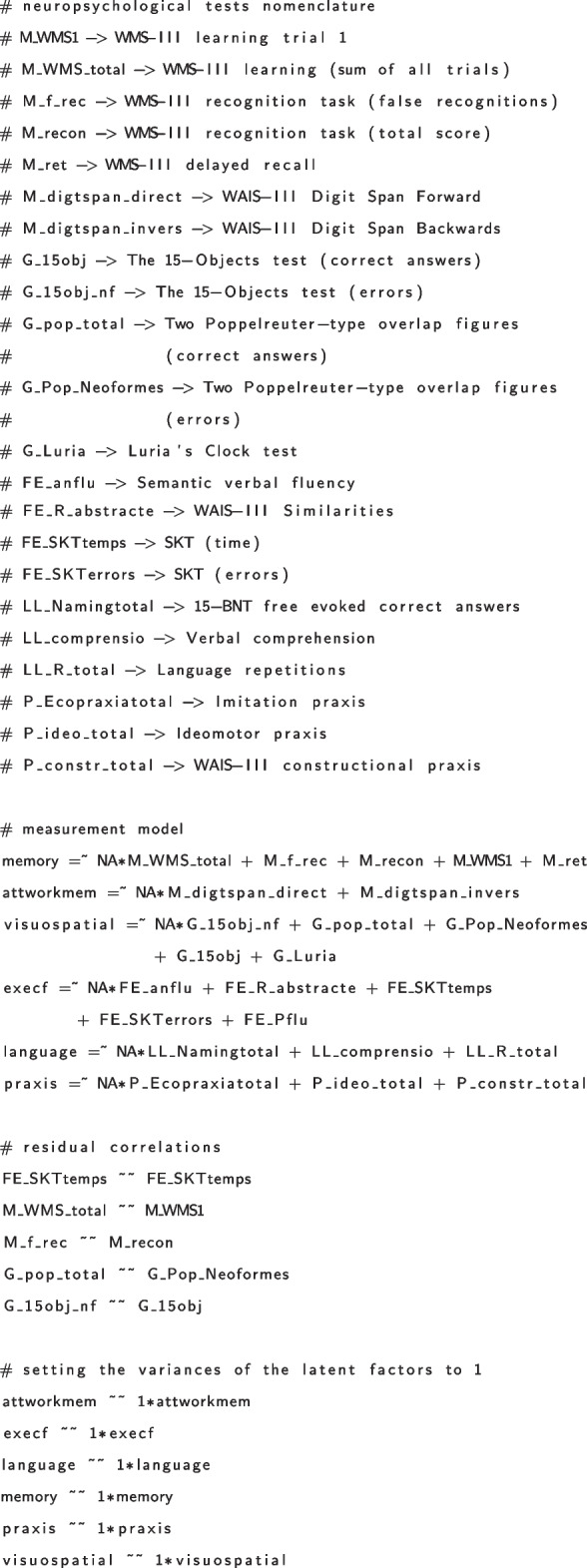
**Listing 1** Syntax (according to the laavan library) used to calculate composite scores

## Data Availability

The datasets generated and/or analyzed during the current study are not publicly available due to they contain human privacy-sensitive data but are available from the corresponding author on reasonable request.

## Appendix A. Calculation of cognitive composites

Machine Learning (ML) models were developed for the prediction of neuropsychological composites. This appendix details the calculation of the composite scores generated from the Neuropsychological Battery from Fundació Ace (NBACE) battery [7].

As mentioned in the main manuscript, the memory composite was created considering the variables long-term and recognition memory of the Word List subtest from the Wechsler Memory Scale, third version (WMS-III) [51]. The attention composite included the Digit Forward and Digit Backwards from the Wechsler Adult Intelligence Scale, Third Edition (WAIS-III) [52]. To define the visuospatial functions, the 15-Objects Test [53], the Poppelreuter-type overlap figures [54], and the Luria’s Clock test [55] were considered. The executive functions were calculated from the Phonetic and Semantic Verbal fluencies [56, 57] and the Automatic Inhibition subtest of the Syndrom Kurtz Test (SKT) [58]. Finally, language function included the abbreviated 15-item naming test from the Boston Naming Test (BNT) [59] and the Verbal Comprehension and Repetitions [7]. The subtest measuring the time taken to complete the SKT was transformed to a logarithmic scale for structural equation models (SEM) fitting.

All available baseline observations collected in Ace were used for the composite scores calculation. Healthy controls (HC) and individuals with subjective cognitive decline (SCD) with a diagnosis of preserved cognition (CDR = 0), patients with mild cognitive impairment (MCI) (CDR = 0.5), and subjects with dementia due to Alzheimer’s disease (ADD) (CDR $\ge$ 1) were selected. Table 5 lists the demographic characteristics of the subjects used to calculate the composite scores. The syntax of the structural equation model used to calculate the composite scores is given in Listing 1. The model fitted with the entire Ace database was subsequently used to infer the composite scores for the sample of subjects used for the study.

**Table 5:** Clinical and sociodemographic characteristics of the sample used to estimate the parameters of the structural equation models used to calculate the composite scores

	All sample	HC/SCD	MCI	ADD
Sex (% females)	67.09	69.73	62.79	71.04
Age (mean (SD))	75.54 (9.93)	64.51 (9.66)	73.85 (9.23)	80.65 (7.05)
Years of formal education (mean (SD))	7.28 (4.60)	11.08 (4.32)	7.54 (4.42)	5.88 (4.18)
MMSE (mean (SD))	24.00 (4.72)	29.13 (1.25)	26.21 (2.94)	20.08 (3.85)

Abbreviations: *SD*, standard deviation; *MMSE*, Mini-Mental State Examination

## Appendix B. Machine Learning model hyperparameters

This appendix details the hyperparameters of all the ML models used in the main manuscript. Table 6 lists the hyperparameters of the algorithms used for the classification and regression problems. The library with the implementation of the genetic algorithm (GA) used is available on GitHub. Table 7 summarizes the hyperparameters of the GA. The Scikit-learn [65] library was used for models: random forest, support vector machines, and k-nearest neighbors. For the XGBoost algorithm, the xgboost [66] library was employed. The hyperparameter optimization (HPO) was carried out using the optuna [62] library. The definition of the hyperparameters listed in the table can be found in the documentation of the respective libraries.

**Table 6:** Hyperparameters of classification and regression models

Model	Parameter^a^	HPO^b^	Value/search space^c^
Random forest	Number of estimators	-	200
	Class weight^d^	-	Balanced
	Max depth	TPE	{2, $\ldots$, 10}
	Min samples split	TPE	{2, $\ldots$, 40}
	Min samples leaft	TPE	{2, $\ldots$, 30}
	Max samples	TPE	[0.5, 1.0]
	Max features	TPE	[0.5, 1.0]
XGBoost	Number of estimators	-	200
	Max depth	TPE	{2, $\ldots$, 10}
	Learning rate	TPE	[0.01, 0.3]
	Gamma	TPE	[0.0, 100.0]
	Min child weight	TPE	[0.0, 100.0]
	Subsample	TPE	[0.2, 1.0]
	colsample_bytree	TPE	[0.2, 1.0]
	colsample_bynode	TPE	[0.2, 1.0]
	L1 regularization	TPE	[0.1, 10.0]
	L2 regularization	TPE	[0.1, 10.0]
	Scale positive weight^d^	TPE	[0.1, 10.0]
Support vector machines	Kernel	-	Polynomial
	C	TPE	[1e-05, 1e02]
	Degree	TPE	{1, $\ldots$, 10}
	Class weight^d^	TPE	[0.05, 0.95]
	Coef_0_	TPE	[0.0, 10.0]
K-nearest neighbors	Number of neighbors	Grid search	{4, $\ldots$, 30}
	Weights	Grid search	{Uniform, Distance}

For the hyperparameter optimization conducted using tree-structured Parzen estimator (TPE), a total of 1000 configurations were sampled, with the initial 500 being randomly selected

^a^Hyperparameters not listed in this table were selected at their default value

^b^Hyperparameters that were left fixed are specified by “-”

^c^$\{\cdot \}$ indicates sampling of categorical values, while $[\cdot ]$ indicates sampling of real values

^d^Parameter only considered for classification problems

**Table 7:** Hyperparameters of the genetic algorithm used to perform feature selection

Parameter	Value	Description
Generations	1,000	Number of algorithm iterations
Population size	300	Number of candidate solutions (aka individuals) in the population
Individual representation	Binary	The candidate solutions were represented as a binary array where a value of 1 indicated the presence of a feature and a value of 0 its absence
Selection	Tournament selection (*k *= 2)	Stochastically, *k* individuals are selected from the whole population. From this selection, the individual with the best fitness value is selected for the next generation. This process is repeated until fill established population size
Elitism	30	Individuals who will pass to the next generation without undergoing the selection process
Mutation	Bit-flip (probability = 0.05)	In each generation, each position of the candidate solution inverts its value according to the specified probability
Cross-over	One-point (probability = 0.5)	In each generation, two individuals are selected with a certain probability and their information is combined by splitting the solution by a certain cut-off point. The resulting fragments are used to generate the offspring

## Appendix C. Cognitive composites analysis

This appendix collects the results of the regression models used to analyze the composites generated in the studied sample. Table 8 contains the values represented in Fig. 2 of the main manuscript.

**Table 8:** Regression model results for the study sample, controlling for sex, age, and years of formal education

Composite	Condition	CDR	Coef.^a^	*p*-value	_95%_CI
Attention	SCD	0.0	1.19	< 0.001	[1.10, 1.29]
	MCI	0.5	− 0.73	< 0.001	[− 0.83, − 0.62]
	Mild ADD	1.0	− 1.15	< 0.001	[− 1.26, − 1.04]
	Moderate ADD	2.0	− 1.47	< 0.001	[− 1.60, − 1.33]
Executive function	SCD	0.0	1.48	< 0.001	[1.39, 1.57]
	MCI	0.5	− 0.91	< 0.001	[− 1.01, − 0.81]
	Mild ADD	1.0	− 1.58	< 0.001	[− 1.69, − 1.48]
	Moderate ADD	2.0	− 1.90	< 0.001	[− 2.03, − 1.77]
Language	SCD	0.0	1.15	< 0.001	[1.05, 1.24]
	MCI	0.5	− 0.63	< 0.001	[− 0.73, − 0.53]
	Mild ADD	1.0	− 1.31	< 0.001	[− 1.42, − 1.20]
	Moderate ADD	2.0	− 1.67	< 0.001	[− 1.81, − 1.54]
Memory	SCD	0.0	1.53	< 0.001	[1.43, 1.62]
	MCI	0.5	− 1.01	< 0.001	[− 1.12, − 0.91]
	Mild ADD	1.0	− 1.78	< 0.001	[− 1.89, − 1.67]
	Moderate ADD	2.0	− 1.95	< 0.001	[− 2.08, − 1.82]
Visuospatial function	SCD	0.0	1.22	< 0.001	[1.12, 1.33]
	MCI	0.5	− 0.73	< 0.001	[− 0.84, − 0.62]
	Mild ADD	1.0	− 1.49	< 0.001	[− 1.61, − 1.37]
	Moderate ADD	2.0	− 1.88	< 0.001	[− 2.02, − 1.73]

Abbreviations: *CDR*, Clinical Dementia Rating; *CI,* confidence interval

^a^Coefficients of the regression models considering the composite value as the dependent variable and the dummy coding of the clinical diagnosis as the independent variable. The models were adjusted for sex, age, and educational level. The coefficients indicate the average value of the composite associated with each group adjusting for the covariates

## Appendix D. Machine Learning model results

This appendix summarizes the results obtained by the classification (Table 9) and regression models (Table 10) applied for the differentiation of clinical phenotypes and the prediction of composite cognitive scores, respectively. For each model, the average results obtained on the test set of a ten-fold cross-validation are shown.

**Table 9:** Average values of the classification metrics computed on the test set for the binary classification problems

Problem	Model	BA	F1	Sen	Spe	Pre
SCD vs altered	RF	0.749	0.854	0.764	0.733	0.967
	XGB	0.739	0.884	0.819	0.659	0.960
	GA-SVM	0.725	0.875	0.805	0.644	0.958
	GA-KNN	0.680	0.742	0.605	0.756	0.961
SCD vs ADD	RF	0.820	0.916	0.900	0.741	0.933
	XGB	0.842	0.920	0.898	0.785	0.944
	GA-SVM	0.830	0.893	0.844	0.815	0.948
	GA-KNN	0.734	0.774	0.661	0.807	0.932
SCD vs MCI	RF	0.692	0.840	0.770	0.615	0.924
	XGB	0.680	0.807	0.715	0.644	0.925
	GA-SVM	0.693	0.841	0.772	0.615	0.925
	GA-KNN	0.628	0.656	0.508	0.748	0.925
MCI vs ADD	RF	0.668	0.614	0.669	0.668	0.569
	XGB	0.670	0.625	0.715	0.625	0.555
	GA-SVM	0.635	0.595	0.704	0.567	0.516
	GA-KNN	0.564	0.532	0.654	0.475	0.449

Abbreviations: *BA*, balanced accuracy; *F1*, f1-score; *RF*, random forest; *XGB*, extreme gradient boosting; *GA-SVM*, genetic algorithm-support vector machine; *GA-KNN*, genetic algorithm-K-nearest neighbors

**Table 10:** Regression metrics obtained in the prediction of the composite cognitive scores

Composite	Model	RMAE (%)	MAE	Correlation	EV (%)
Attention	RF	18.0	0.474	0.468	21.8
	XGB	18.0	0.475	0.464	21.6
	GA-SVM	18.8	0.495	0.407	14.9
	GA-KNN	19.2	0.506	0.352	11.4
Executive function	RF	17.7	0.486	0.552	30.1
	XGB	17.4	0.477	0.563	31.7
	GA-SVM	18.3	0.502	0.517	26.1
	GA-KNN	19.2	0.527	0.450	19.9
Language	RF	18.1	0.466	0.548	29.9
	XGB	17.8	0.458	0.569	32.3
	GA-SVM	19.7	0.508	0.493	21.4
	GA-KNN	19.9	0.511	0.418	17.1
Memory	RF	18.5	0.534	0.494	24.3
	XGB	18.3	0.530	0.499	24.9
	GA-SVM	19.6	0.568	0.407	14.9
	GA-KNN	20.2	0.584	0.354	10.2
Visuospatial function	RF	18.1	0.521	0.565	31.7
	XGB	17.9	0.515	0.574	33.0
	GA-SVM	20.1	0.578	0.498	22.3
	GA-KNN	20.0	0.577	0.430	18.1

Abbreviations: *MAE*, mean absolute error; *RMAE*, relative MAE (described in the “Experimental setup” section); *EV*, explained variance; *RF*, random forest; *XGB*, extreme gradient boosting; *GA-SVM*, genetic algorithm- support vector machine; *GA-KNN*, genetic algorithm-K-nearest

## Appendix E. Features selected by the genetic algorithms

This appendix collects the features selected by the models that underwent a preliminary feature selection step using GAs (i.e., SVM and KNN). Table 11 shows the percentage of times in which each variable was selected in each fold for each problem. To simplify the information, only variables consistently chosen across different folds are included, encompassing those that appeared more than 75% of the time in each of the problems examined in the study. The total number of features selected in each of the folds by each of the algorithms in each problem is provided in a separate Excel document (see *Selected-features-GA.xlsx*).

**Table 11:** Percentage of times each feature was selected across cross-validation folds for the different problems by the genetic algorithms

Feature	P1	P2	P3	P4	P5	P6	P7	P8	P9
F0	100	100	100	100	100	100	100	100	100
F1	100	100	100	-	100	100	100	100	100
F2	100	100	100	-	100	100	95	100	90
F3	100	100	100	-	100	95	90	-	95
F4	-	-	-	-	100	90	75	95	90
F5	-	100	-	100	-	-	85	80	80
F6	75	100	80	85	-	-	-	-	85
F7	75	-	-	-	-	85	75	95	90
F8	-	-	80	-	85	75	-	90	80
F9	-	-	-	-	80	95	95	-	85
F10	-	-	-	-	-	85	95	90	80
F11	-	-	-	-	75	85	80	-	95
F12	-	-	-	-	90	75	-	75	95
F13	-	-	-	-	-	75	80	90	85
F14	-	75	-	-	-	80	-	85	80
F15	95		100	85	-	-	-	-	-
F16	95	75	100	-	-	-	-	-	-
F17	-	-	-	-	80	-	-	75	90
F18	-	-	-	90	-	-	80	75	-
F19	-	85	-	-	-	-	80	-	75
F20	75	-	-	-	-	75	85	-	-
F21	-	-	-	-	75	-	80	-	75
F22	75	-	-	-	-	80	75	-	-
F23	-	80	-	100	-	-	-	-	-
F24	-	-	-	-	90	-	85	-	-
F25	-	-	-	80	-	-	90	-	-
F26	-	-	-	-	-	-	-	80	90
F27	-	-	-	80	-	-	90	-	-
F28	-	-	-	90	-	-	80	-	-
F29	-	-	-	-	-	75	90	-	-
F30	-	-	-	-	90	-	75	-	-
F31	-	-	-	-	-	-	-	75	90
F32	75	85	-	-	-	-	-	-	-
F33	-	-	80	80	-	-	-	-	-
F34	-	-	-	-	80	-	-	-	75
F35	80	-	75	-	-	-	-	-	-
F36	75	-	-	-	-	75	-	-	-
F37	-	75	-	-	-	-	75	-	-
F38	75	75	-	-	-	-	-	-	-
F39	-	75	75	-	-	-	-	-	-
F40	-	-	-	-	100	-	-	-	-
F41	-	95	-	-	-	-	-	-	-
F42	-	-	-	-	90	-	-	-	-
F43	-	-	-	-	-	-	-	-	90
F44	-	-	-	-	-	-	90	-	-
F45	-	-	-	-	-	-	85	-	-
F46	85	-	-	-	-	-	-	-	-
F47	-	-	-	-	-	-	-	85	-
F48	-	-	-	-	85	-	-	-	-
F49	-	-	85	-	-	-	-	-	-
F50	-	-	-	-	-	-	80	-	-
F51	-	-	-	-	-	80	-	-	-
F52	-	-	-	-	-		-	80	-
F53	-	-	-	-	-	80	-	-	-
F54	-	-	-	-	-	-	80	-	-
F55	-	-	-	80	-	-	-	-	-
F56	-	-	-	-	-	-	-	-	80
F57	-	-	-	-	80	-	-	-	-
F58	-	-	-	-	80	-	-	-	-
F59	-	-	80	-	-	-	-	-	-
F60	-	-	-	80	-	-	-	-	-
F61	-	-	-	80	-	-	-	-	-
F62	-	-	-	-	80	-	-	-	-
F63	-	-	80	-	-	-	-	-	-
F64	-	-	-	-	-	-	-	-	80
F65	-	-	-	-	-	-	-	-	75
F66	-	-	-	-	-	-	-	75	-
F67	-	-	-	-	-	-	75	-	-
F68	-	-	-	-	-	-	75	-	-
F69	-	-	-	-	-	-	-	75	-
F70	-	-	-	-	-	-	75	-	-
F71	-	-	-	-	-	-	-	75	-
F72	-	-	-	-	75	-	-	-	-
F73	-	-	-	-	-	75	-	-	-
F74	-	-	-	-	-	75	-	-	-
F75	-	-	-	-	75	-	-	-	-
F76	-	-	-	-	75	-	-	-	-
F77	-	-	-	-	75	-	-	-	-
F78	-	-	-	-	75	-	-	-	-
F79	75	-	-	-	-	-	-	-	-
F80	75	-	-	-	-	-	-	-	-
F81	75	-	-	-	-	-	-	-	-
F82	-	75	-	-	-	-	-	-	-
F83	-	-	75	-	-	-	-	-	-
F84	-	-	75	-	-	-	-	-	-
F85	-	75	-	-	-	-	-	-	-
F86	-	-	-	-	-	-	-	-	75

^a^The names of the features are listed in Table 12

Abbreviations: *P1*, SCD vs cognitive impairment; *P2*, SCD vs ADD; *P3*, SCD vs MCI; *P4*, MCI vs ADD; *P5*, attention; *P6*, executive function; *P7*, memory; *P8*, language; *P9*, visuospatial function

**Table 12:** Abbreviations of the feature names listed in Table 11

Code^a^	Feature name	Code^a^	Feature name
F0	Age	F44	(AE) Frequency F2-bandwidth (voiced) AMean
F1	Years of formal education	F45	(ID) Frequency F0-semitonefrom-27.5hz (voiced) 20-80th percentile range
F2	(AE) Spectral flux (voiced) CoV	F46	(AE) Frequency F3-bandwidth (voiced) CoV
F3	(ID) Energy/Amplitude Loudness CoV	F47	(ID) Frequency F2-amplitudelogrelf0 (voiced) AMean
F4	(ID) Temporal-feature Loudness-Peak/second	F48	(ID) Frequency F1-frequency (voiced) CoV
F5	(AE) Ceptral MFCC4 (voiced) CoV	F49	(ID) Ceptral MFCC4 CoV
F6	(AE) Frequency F0-semitonefrom-27.5hz (voiced) Mean rising slope	F50	(AE) Spectral Alpharatio (voiced) CoV
F7	(ID) Energy/Amplitude Shimmer-localdb (voiced) CoV	F51	(ID) Frequency F0-semitonefrom-27.5hz (voiced) Std falling slope
F8	(AE) Frequency F0-semito nefrom-27.5hz (voiced) CoV	F52	(AE) Frequency F3-frequency (voiced) CoV
F9	(AE) Spectral flux (unvoiced) Amean	F53	(AE) Ceptral MFCC3 (voiced) CoV
F10	(AE) Frequency F0-semitonefrom-27.5hz (voiced) 20-80th percentile range	F54	(AE) Spectral Hammarberg index (unvoiced) AMean
F11	(ID) Energy/Amplitude Loudness 20th percentile	F55	(AE) Ceptral MFCC2 CoV
F12	Energy/Amplitude Loudness 50th percentile	F56	Frequency F0-semitonefrom-27.5hz (voiced) 20th percentile
F13	Spectral flux (unvoiced) Amean	F57	Spectral Hammarberg index (unvoiced) AMean
F14	Spectral Alpharatio (unvoiced) AMean	F58	(AE) Energy/Amplitude Loudness Mean falling slope
F15	(AE) Spectral Slope500-1500 (unvoiced) Amean	F59	(AE) Frequency F0-semitonefrom-27.5hz (voiced) Std rising slope
F16	(AE) Frequency F3-bandwidth (voiced) AMean	F60	(AE) Spectral Slope0-500 (unvoiced) Amean
F17	Temporal-feature Unvoiced-Segment-Length/second Mean	F61	(AE) Ceptral MFCC4 AMean
F18	(AE) Frequency F0-semitonefrom-27.5hz (voiced) Mean falling slope	F62	(AE) Ceptral MFCC1 (voiced) AMean
F19	(AE) Frequency F2-bandwidth (voiced) CoV	F63	(AE) Frequency F2-frequency (voiced) CoV
F20	(AE) Frequency F2-frequency (voiced) AMean	F64	Frequency F2-bandwidth (voiced) AMean
F21	Frequency F0-semitonefrom-27.5hz (voiced) CoV	F65	Frequency F3-amplitudelogrelf0 (voiced) AMean
F22	(AE) Spectral Slope500-1500 (voiced) AMean	F66	Frequency F0-semitonefrom-27.5hz (voiced) Mean rising slope
F23	(AE) Temporal-feature Voiced-Segment-Length/second Mean	F67	Spectral flux (voiced) CoV
F24	Spectral Slope500-1500 (voiced) CoV	F68	(AE) Energy/Amplitude Loudness 20-80th percentile range
F25	(AE) Spectral Alpharatio (unvoiced) AMean	F69	(ID) Spectral flux AMean
F26	(AE) Ceptral MFCC1 CoV	F70	(AE) Ceptral MFCC4 (voiced) AMean
F27	(AE) Spectral Harmonic difference H1-H2 (voiced) CoV	F71	(AE) Energy/Amplitude Loudness Std rising slope
F28	(AE) Frequency F0-semitonefrom-27.5hz (voiced) Std falling slope	F72	Spectral Slope500-1500 (unvoiced) Amean
F29	(AE) Energy/Amplitude Loudness 50th percentile	F73	(AE) Spectral flux AMean
F30	(AE) Energy/Amplitude Loudness Mean rising slope	F74	Energy/Amplitude Shimmer-localdb (voiced) AMean
F31	Frequency F3-frequency (voiced) CoV	F75	(AE) Others Equivalent-Sound-Level (dB)
F32	Ceptral MFCC1 (voiced) CoV	F76	Temporal-feature Voiced-Segments/second
F33	(AE) Frequency Jitter-local (voiced) CoV	F77	(AE) Energy/Amplitude Loudness AMean
F34	Spectral Slope500-1500 (voiced) AMean	F78	Frequency F2-frequency (voiced) AMean
F35	(AE) Spectral Slope0-500 (voiced) CoV	F79	Frequency F3-bandwidth (voiced) AMean
F36	(AE) Temporal-feature Loudness-Peak/second	F80	(ID) Ceptral MFCC3 AMean
F37	Ceptral MFCC1 (voiced) AMean	F81	(AE) Ceptral MFCC1 (voiced) CoV
F38	Ceptral MFCC3 (voiced) AMean	F82	Frequency F0-semitonefrom-27.5hz (voiced) 50th percentile
F39	Ceptral MFCC4 (voiced) CoV	F83	(AE) Frequency F1-frequency (voiced) AMean
F40	Ceptral MFCC4 (voiced) AMean	F84	(AE) Spectral Harmonic difference H1-H2 (voiced) AMean
F41	(AE) Energy/Amplitude Shimmer-localdb (voiced) CoV	F85	(ID) Ceptral MFCC2 CoV
F42	(AE) Energy/Amplitude Loudness Std falling slope	F86	Frequency F0-semitonefrom-27.5hz (voiced) Std rising slope
F43	Frequency F2-frequency (voiced) CoV		

^a^Feature code used to identify the variable in Table 11

Abbreviations: *ID*, image description task; *AE*, animal enumeration task; *MFCC*, mel-frequency cepstral coefficient; *AMean*, arithmetic mean; *CoV*, coefficient of variation; *HNR*, harmonics-to-noise ratio; *Std*, standard deviation
